# Cardiovagal Baroreflex Hysteresis Using Ellipses in Response to Postural Changes

**DOI:** 10.3389/fnins.2021.720031

**Published:** 2021-12-09

**Authors:** Babak Dabiri, Joana Brito, Eugenijus Kaniusas

**Affiliations:** Institute of Electrodynamics, Microwave and Circuit Engineering, Vienna University of Technology, Vienna, Austria

**Keywords:** cardiovagal baroreflex hysteresis, baroreflex sensitivity, ellipse, autonomic nervous system, orthostatic

## Abstract

The cardiovagal branch of the baroreflex is of high clinical relevance when detecting disturbances of the autonomic nervous system. The hysteresis of the baroreflex is assessed using provoked and spontaneous changes in blood pressure. We propose a novel ellipse analysis to characterize hysteresis of the spontaneous respiration-related cardiovagal baroreflex for orthostatic test. Up and down sequences of pressure changes as well as the working point of baroreflex are considered. The EuroBaVar data set for supine and standing was employed to extract heartbeat intervals and blood pressure values. The latter values formed polygons into which a bivariate normal distribution was fitted with its properties determining proposed ellipses of baroreflex. More than 80% of ellipses are formed out of nonoverlapping and delayed up and down sequences highlighting baroreflex hysteresis. In the supine position, the ellipses are more elongated (by about 46%) and steeper (by about 4.3° as median) than standing, indicating larger heart interval variability (70.7 versus 47.9 ms) and smaller blood pressure variability (5.8 versus 8.9 mmHg) in supine. The ellipses show a higher baroreflex sensitivity for supine (15.7 ms/mmHg as median) than standing (7 ms/mmHg). The center of the ellipse moves from supine to standing, which describes the overall sigmoid shape of the baroreflex with the moving working point. In contrast to regression analysis, the proposed method considers gain and set-point changes during respiration, offers instructive insights into the resulting hysteresis of the spontaneous cardiovagal baroreflex with respiration as stimuli, and provides a new tool for its future analysis.

## Introduction

Blood pressure in humans is governed by the arterial and cardiopulmonary baroreflex, an essential part of the autonomic nervous system. Here, changes in pressure, e.g., spontaneous due to breathing or artificial due to vasoactive drug administration, act as stimuli on baroreceptors. The subsequent changes in heart rate, stroke volume, and total peripheral resistance following changes in the parasympathetic and sympathetic nervous systems compose the reflex response, which, in fact, aims to buffer changes in pressure (Kaniusas, 2012). In particular, sequences of pressure values and the associated heart rate values due to efferent vagal activity to the sinoatrial node form the cardiovagal baroreflex hysteresis loop (deBoer et al., 1987; Bertinieri et al., 1988; Parati et al., 1988; Turjanmaa et al., 1990; Saul et al., 1991).

Chapleau et al. (1988) show, in animals, that a change in static pressure levels induces a dominant sigmoidal relationship between the pressure and baroreceptor activity (of the carotid sinus), whereas this relationship is almost linear for the pulsatile pressure (Chapleau and Abboud, 1987). A linear regression over the linear portion of the sigmoidal function between systolic pressure and heart rate values determines the cardiovagal baroreflex gain, known also as baroreflex sensitivity (BRS; deBoer et al., 1987; Parati et al., 1988; Turjanmaa et al., 1990). A reduced BRS value *BRS* is usually associated with cardiovascular failure (La Rovere et al., 2008), orthostatic intolerance (Cooper and Hainsworth, 2002), neurally mediated syncope (Benarroch, 2008), and amplified pain perception (Suarez-Roca et al., 2019).

For rising and falling systolic pressure values, an elliptical shape of the baroreflex hysteresis becomes dominant (Chapleau and Abboud, 1987; Eckberg and Sleight, 1992; Rudas et al., 1999). The baroreceptor activity seems to be stronger for downward pressure changes as compared with upward pressure changes (Chapleau and Abboud, 1987). However, comparisons of *BRS* for upward and downward pressure changes yield controversial results; e.g., upward changes yield higher *BRS* in Studinger et al. (2007), whereas no differences are observed in De Maria et al. (2019). A spontaneous cardiovagal baroreflex cycle (CBC) results in a course of spontaneous and periodic breathing, which is composed of rise-to-fall and/or fall-to-rise pressure sequences with the associated heart rate values. The presence of hysteresis and varying baroreceptor activity highlight the necessity to consider directional changes of both pressure and heart rate values in terms of the fitted CBC.

To investigate large parts of nonlinear and asymmetrical hysteresis, a wide range of (spontaneous or induced) pressure changes is needed as usually can be used only in animal studies (Chapleau and Abboud, 1987) and also in humans with vasoactive substances (Rudas et al., 1999; Studinger et al., 2007). Small changes of pressure would mainly disclose only the linear parts of the hysteresis near the working point. To investigate the baroreflex, common autonomic provocations include orthostatic tests, head up tilt tests, neck chamber testing, handgrip tests, and the Valsalva maneuver (Hainsworth, 1998; Convertino, 2001; La Rovere et al., 2008; Hart et al., 2011; Ichinose and Nishiyasu, 2012; De Maria et al., 2019; Incognito et al., 2019) as well as pharmacologic interventions (La Rovere et al., 1998; Ler et al., 2010; Hart et al., 2011; Taylor et al., 2013; Incognito et al., 2020). Here, the provoked pressure changes provide a possibility to extract the sympathetic outflow of the autonomic nervous system (e.g., by recording sympathetic nerve activity to the blood vessels) and the corresponding parasympathetic vagal outflow (e.g., by recording heart periods) in terms of baroreflex regulation (Saul et al., 1991; Hart et al., 2011; Taylor et al., 2013).

For instance, the orthostatic stress as a simple, noninvasive test, reduces the spontaneous *BRS* from supine to standing (from 17.5 to 7.65 ms/mmHg; Steptoe and Vögele, 1990) by elevating the vascular sympathetic outflow in response to gravitationally relocated blood volume as assessed by studies without considering the directional change in pressure and, thus, without considering hysteresis. Vasoactive substances (to increase and/or decrease blood pressure) were used to investigate the mechanical arm of the baroreflex over a wide range of pressure changes, reflecting the relationship between the systolic blood pressure and the carotid artery diameter, known as the modified Oxford method (Hunt et al., 2001; Studinger et al., 2007; Ler et al., 2010; De Maria et al., 2019). Here, the coinvestigated neural arm reflects the associated changes in heart rate and carotid artery diameter (Smyth et al., 1969).

From a processing perspective, the morphology of the (spontaneous and nonspontaneous) CBC hysteresis is characterized using quantitative and qualitative parameters. Variability of up and down baroreflex sequences were investigated for orthostatic test and different age (Rudas et al., 1999; Taylor et al., 2013; De Maria et al., 2019). Studinger et al. (2007) show how the baroreflex resets the next heart period solely by the neural arm with the vasoactive substance administration process. Mechanical and neural baroreflex arms were investigated for continuous blood pressure changes (Studinger et al., 2007; Taylor et al., 2013, 2014). Whereas *BRS* for rising and falling blood pressure (e.g., 12 ms/mmHg during supine versus 7.5 ms/mmHg during treadmill activity; De Maria et al., 2019) using vasoactive substances is usually considered in quantitative terms, an alteration in the working point on the hysteresis is usually subjected to qualitative analysis only (Studinger et al., 2007). Independent up and down sequences were assessed without considering their succession in time and, thus, neglecting the neuronal arm accounting for respiration-related directional pressure changes from one sequence to another (Bertinieri et al., 1985; Steptoe and Vögele, 1990; De Maria et al., 2018, 2019). A bivariate phase-rectified signal averaging technique was used to quantify upward and downward sequences (De Maria et al., 2018). A 3-D planar ellipse method was introduced to describe both arms of the baroreflex using vasoactive substances (Ler et al., 2010).

In this work, we propose a novel approach using sophisticated ellipse analysis to characterize cardiovagal baroreflex hysteresis on two levels: (i) cardiovagal hysteresis due to the respiration-related spontaneous pressure change (i.e., not pharmacologically induced), and (ii) cardiovagal hysteresis due to the static pressure change in the course of the orthostatic test. In contrast to the state of the art, we separately consider the up and down sequences of spontaneous pressure changes (for expiration and inspiration, respectively), which show individual values of *BRS* and then compose a full ellipse (for a whole respiratory cycle) with its own *BRS*. This BRS accounts for all three: mechanical arm, neural arm acting within single sequences, and neuronal arm accounting for directional pressure changes (set-point changes). Spontaneous and intrinsic regulatory processes are considered only without any artificial pressure perturbation and without the associated cofactors/interferences affecting barosensory vessel mechanics. In addition, we analyze quantitatively the working point changes of the baroreflex during the orthostatic test using our ellipse-based analysis. Here, we employ the maneuver of the change in posture to vary the static blood pressure in a binary way and, thus, to characterize the position and change of the working point and the sigmoidal behavior of the CBC.

## Materials and Methods

This study was performed on the EuroBaVar data set^1^, provided by the Working Group on Blood Pressure and Heart Rate Variability of the European Society of Hypertension. The electrocardiogram data consists of 42 recordings of 21 subjects (17 females, median age = 38.4 years, median height = 1.65 m, median weight = 64.1 kg, and median body mass index = 23.3 kg/m^2^), including both series of recordings, series A (*n* = 8) and series B (*n* = 13), and both positions, supine and upright, each position lasting for 10–12 min. Informed consent was obtained, and the study was approved by the Paris–Necker committee for the protection of human subjects in biomedical research. Study subjects were composed of 12 normotensive, 4 healthy, 3 hypertensive (1 treated with medication and 2 nonmedicated), one diabetic, and one heart transplantation patients. The detailed data can be found in Laude et al. (2004) and Choi et al. (2006). Noninvasive monitoring devices were used such as a three-lead electrocardiogram (Cardiocap II; Datex Engstrom, Helsinki, Finland) and a continuous beat-to-beat blood pressure monitor (Finometer MIDI; Finapres Inc., Enschede, Netherlands) with all signals recorded at a 500-Hz sampling rate.

The respiratory signal was not recorded. However, we assume that up and down sequences in each CBC are due to the respiratory sinus arrhythmia and correspond to expiration and inspiration phases, respectively (Silva et al., 2019). This is because other nonrespiratory periodic modulating mechanisms in the frequency region of the respiration were not present during recordings.

In the recorded data, *RR*-intervals of the duration *RR*, the systolic pressure *P*_S_, and the diastolic pressure *P*_D_ were identified and verified visually. Up and down baroreflex sequences with three or more beats, i.e., in which *P*_S_ and *RR* values progressively increased or decreased, were extracted in all recordings. Only those *P*_S_ and *RR* sequences were considered that had a mutual correlation coefficient >0.85, i.e., the sequences that were assumed to reflect predominantly the cardiovagal *BRS* (Hughson et al., 1993). There was no minimum *P*_S_ change required for the sequence to be valid. If an up sequence was immediately followed by a down sequence or vice versa, both sequences were considered as CBC. Consequently, for *n* consecutive up and down sequences (with *n* > 2), we end up with (*n* −1) CBCs.

**FIGURE 1:**
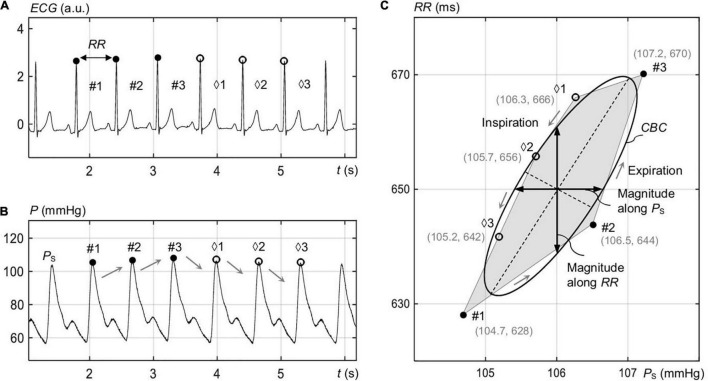
Identification of the spontaneous CBC in the course of respiration for elliptic processing. **(A)** Electrocardiogram signal ECG with rising *RR* values for the marked intervals #1 to #3 (expiration) and falling *RR* for the intervals ♢1 to ♢3 (inspiration). **(B)** The blood pressure *P* with the associated rising and falling systolic pressure *P*_S_ values. **(C)** Vertices with filled dots correspond to rising *P*_S_ and *RR*, and empty dots correspond to falling *P*_S_ and *RR*. The shown ellipse represents the overall ellipse of the image-based procedure.

As illustrated in Figure 1C, the resulting CBC as a function of *P*_S_ (*x*-axis) and *RR* (*y*-axis) shows a polygon shape. Here, the filled points represent the up sequence, and the empty points represent the down sequence.

### Ellipse Analyses for Nonintersecting Sequences

To approximate CBC—composed of nonintersecting sequences only—with ellipses, we identified them in the 2-D area (encompassed by *x*- and *y*-axes) in a way that ellipses enclose the whole polygon while exhibiting the minimum area. For this, an iterative procedure of the convex minimization problem, known as the Khachiyan algorithm (Kumar and Yildirim, 2005), was employed.

For the ellipses to show the same area as the original polygons, we linearly downscaled these resulting ellipses with respect to their centers but without changing their orientation angle with respect to the *x*- and *y*-axes. Here, the center of the ellipse, its minor and major axes, as well as the orientation angle comprise the representative parameters of CBC.

### Ellipse Analysis for all Sequences

In the case that up and down sequences have one or more intersection points as it is often the case (see Supplementary Material), multiple closed polygons necessarily appear (Supplementary Figure 1). Because the aforementioned method considers only nonintersecting sequences, we estimate the best fitted ellipse for both nonintersecting and intersecting sequences using a proposed image-based procedure.

First, we cropped the 2-D area of the prospective CBC with the pixel resolution of 0.1 mmHg × 0.1 ms from the minimum values of *P*_S_ (=75 mmHg) and *RR* (=500 ms) of all recordings to the respective maximum values (175 mmHg and 1,500 ms) of all recordings. Thus, the resulting image had a total resolution of 1,000 × 10,000 px^2^. The image was binarized with black color outside of the polygon and white color inside of it (Supplementary Figure 1).

Then, a bivariate normal distribution is fitted into each closed polygon within the generated binary image. The covariance matrix of the distribution determines the shape of the distribution. The contour lines of each distribution form an ellipse. In particular, the direction and length of the major and minor axes of the ellipse for each closed polygon are given by eigenvectors and eigenvalues of the covariance matrix. The orientation angle of the respective closed polygon is given by the first eigenvector and represents the respective angle between the major axis and the *x*-axis. The intersection of the major and minor axes determines the weighted center of the ellipse shape (for each closed polygon).

Consequently, we end up with as many ellipses as closed polygons. The area *A*_O_ of the overall ellipse for CBC is defined as the sum of respective individual areas *A*_I_ of the closed polygons, whereas the center coordinates (*x*_O_, *y*_O_) of the overall ellipse and its orientation angle θ_O_ resulted out of a weighting procedure considering individual ellipses with their center coordinates (*x*_*I*_, *y*_*I*_) and individual orientation angles θ_I_ for each closed polygon to give


(1) $$x_{\text{O}}=\frac{\sum x_{\text{I}}\cdot A_{\text{I}}}{\sum A_{\text{I}}},y_{\text{O}}=\frac{\sum y_{\text{I}}\cdot A_{\text{I}}}{\sum A_{\text{I}}},\theta_{\text{O}}=\frac{\sum \theta_{\text{I}}\cdot A_{\text{I}}}{\sum A_{\text{I}}},\text{and}\,A_{\text{O}}=\sum A_{\text{I}}.$$

Because the individual closed polygons show comparable values of θ_*I*_ in the range of ±1%, the total length *l*_O_MAJOR_ of the major axis of the overall ellipse is simply estimated as the sum of respective lengths *l*_*I_MAJOR*_ of major axes of all individual closed polygons. The total length *l*_O_MINOR_ of the minor axis for CBC resulted out of a weighting procedure of individual *l*_*I_MINOR*_—in analogy with Eq. 1—to give


(2) $$l_{\text{O}\,\_\,\text{MINOR}}=\frac{\sum l_{\text{I}\,\_\,\text{MINOR}}\cdot A_{\text{I}}}{\sum A_{\text{I}}}\,\text{and}\,l_{\text{O}\,\_\,\text{MAJOR}}=\sum l_{\text{I}\,\_\,\text{MAJOR}}.$$

All formed individual and overall ellipses are visually controlled to encompass closed polygons. The value of *BRS* for CBC is estimated out of θ_O_ based on


(3) $$B\,R\,S=\tan^{-1}\theta_{\text{O}}.$$

To quantitatively assess the share of intersecting and nonintersecting up and down sequences forming hysteresis of CBC, each sequence is fitted individually with a subellipse (as illustrated within the inset of Figure 8 by dashed subellipses). A ratio *a*/(*b*/2) is built of the minor axis (=*a*) of each subellipse to the half of the minor axis (=*b*/2) of the overall CBC ellipse. Nonoverlapping sequences show *a*/(*b*/2) ≤ 1, whereas those with an overlap show *a*/(*b*/2) > 1.

### Regression Method

Linear regression analysis was applied to the selected sequences of *P*_S_ and *RR*. Ascending and descending trends were separately assessed. For the linear regression analysis, the sequences of *P*_S_ and *RR* were used without any delay in between (Figure 1C; Steptoe and Vögele, 1990).

### Statistics

A paired *t*-test was used to compare the mean *BRS* and the mean magnitudes of the hysteresis (defined as the maximum change in *RR* for a constant *P*_S_) as well as to compare *P*_S_, *P*_*D*_, and *RR* between supine and standing positions. A Wilcoxon signed rank test was used to compare the median *BRS* values between supine and standing positions. The differences were considered statistically significant when the error probability *p* < 0.05. Data are presented as median and/or interquartile range (IQR), supplemented by mean ± standard deviation. All data were processed and analyzed in MATLAB R2020b (The MathWorks Inc., Natick, MA, United States).

## Results

### Beat-to-Beat Analysis for Respiration

Figure 1 illustrates consecutive up and down sequences of *RR* (Figure 1A) and *P*_S_ (Figure 1B) in the course of respiration for a single recording, forming a spontaneous CBC (Figure 1C). In fact, the up sequence reflects expiration, whereas the down sequence reflects inspiration, in line with the process of respiratory sinus arrythmia. An ellipse is shown in Figure 1C that approximates a closed polygon, composed of P intervals and *P*_S_ values for the points #1 to #3 (up sequence) and ♢1 to ♢3 (down sequence) as estimated by the image-based procedure. The median respiration rate for standing was 12.6 (12.7 ± 2) 1/min, whereas that for supine was 11.1 (11.3 ± 1.9) 1/min.

### Beat-to-Beat Analysis for Postural Changes

**FIGURE 2:**
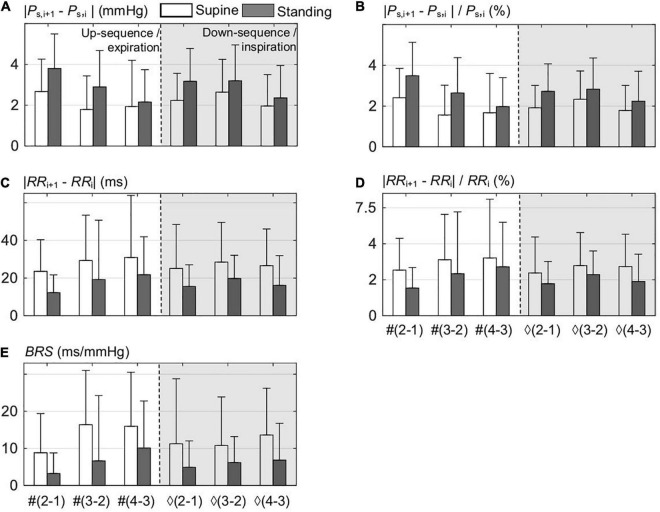
Beat-to-beat analysis of up sequences composed out of *i*th points, i.e., from #1 to #3, and down sequences out of ♢1 to ♢3 points; both sequences compose the spontaneous CBC for supine and standing. **(A)** The differences between the consecutive (*i*+1) and *i* values within the sequence of the systolic blood pressure *P*_S_. **(B)** Normalized differences of **(A)**. **(C)** The differences between the consecutive (*i*+1) and *i* values within the sequence of *RR* values. **(D)** Normalized differences of **(C)**. **(E)** The associated values of the baroreflex sensitivity *BRS* for all differences.

A detailed beat-to-beat analysis of up and down sequences composing the spontaneous CBC is shown in Figure 2 for supine and standing. In particular, the beat-to-beat deflections of *P*_S_ (Figure 2A) and *RR* (Figure 2C) illustrate that the maximum *P*_S_ deflection occurs at the onset of expiration in both supine and standing. The standing position has a higher deflection in *P*_S_ and a lower one in *RR* than the supine position, which already indicates a higher baroreflex efficiency in supine with stronger smoothed changes in *P*_*S.*_ In particular, the mean deflection in *P*_S_ in standing amounts to about ±2.4% and in supine to ±1.8% (Figure 2B) considering single beat-to-beat deflections, whereas the mean deflection in *RR* follows a reverse trend, i.e., in standing, it amounts to about ±2.5%, whereas in supine to ±3% (Figure 2D).

Figure 2E shows that the mean value of *BRS* for standing is only about half of that in supine (=53%), which again highlights the higher baroreflex efficiency in supine. It is instructive to observe that the magnitude of deflections in *P*_S_ is inversely related to the values of *BRS* and is proportional to those in *RR*, which confirms the buffering activity of baroreflex—proportional to *BRS*—with respect to *P*_S_ at the cost of *RR* variability (Figures 2B,D). Please note that the lowest value of *BRS* results at the first two beats of up sequences, i.e., at the start of expiration.

### Ellipse Analysis for Postural Changes

**FIGURE 3:**
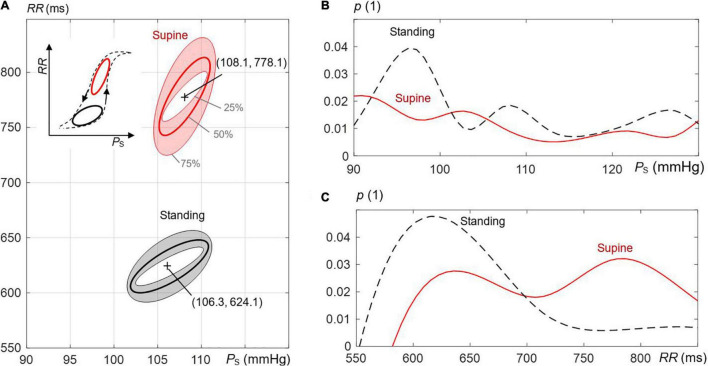
The effect of postural changes on the estimated ellipses of the spontaneous CBC in the course of respiration. **(A)** Supine versus standing is illustrated; the shaded regions mark interquartile ranges, and bold lines mark median values. Inner and outer ellipses represent, respectively, the 25th to 75th interquartile, and the middle ellipse is the median ellipse. The supine position displays more elongated and steeper hysteresis (with respect to the axis of the systolic pressure *P*_S_) than the standing position. The inset to the left shows the schematic behavior of the whole hysteresis, including both postural positions. The frequency distributions of **(B)** *P*_S_ and **(C)** RR-values *RR* for supine and standing with *p* as the probability.

Figure 3A compares ellipses for supine and standing for all recordings, including median and IQR ellipses of all estimated (overall) ellipses based on an image-based procedure. The distributions of *RR* and *P*_S_ are illustrated in Figures 3B,C for supine and standing. In fact, the median values of these distributions determine the center coordinates of median ellipses (Figure 3A). For visual simplicity, IQR ellipses in Figure 3A are relocated in a way that their centers overlap with the center of the associated median ellipses. As expected, the angle θ_O_ increases from the 25th to the 75th quantile as also confirmed by quantitative data in Table 1.

**TABLE 1:** Characteristic parameters of the overall ellipses of spontaneous cardiovagal baroreflex cycles for supine and standing (Figure 3).

	Major axis length *c*_O_MAJOR_*		Minor axis length *c*_O_MINOR_*		Orientation angle θ_O_ (°)	
	Supine	Standing	Supine	Standing	Supine	Standing
25th quantile (inner ellipse)	45.6	35.8	2.4	3.8	83.9	79.0
Median (middle ellipse)	70.8	48.4	3.6	5.8	85.8	81.5
75th quantile (outer ellipse)	107	65.2	5.8	8.0	87.4	84.6

*All differences between supine and standing are statistically significant (p < 0.05).*

*(mmHg^2^ + ms^2^)^1/2^.

In the supine position, the ellipses are more elongated and steeper than in standing (Figure 3A). Quantitative data in Table 1 confirms that *l*_O_MAJOR_ and θ_O_ are statistically larger, whereas *l*_O_MINOR_ is statistically smaller for supine than standing. For supine and standing positions, the median of *l*_O_MAJOR_ is 70.8 (IQR 61.4) and 48.4 (IQR 29.4), whereas the median of *l*_O_MINOR_ is 3.6 (IQR 3.4) and 5.8 (IQR 4.2), respectively. In particular, the median of θ_O_ amounts to 85.8° for supine, which implies that the median ellipse is steeper by 4.3° than for standing. The resulting center of concentric ellipses (median and IQR ellipses) for supine is located at (108.1 mmHg, 778.1 ms), i.e., at higher values than for standing with its coordinates (106.3 mmHg, 624.1 ms).

**FIGURE 4:**
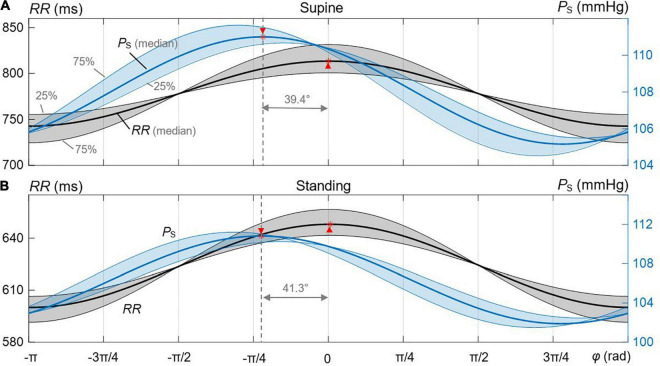
Derived courses of *P*_S_ and *RR* out of the estimated median and IQR ellipses for the spontaneous CBC. The shaded regions represent the 25th and 75th interquartile ranges. **(A)** Supine. **(B)** Standing. The respective maxima and delays are marked between median-related courses of *P*_S_ and *RR.*

Figure 4 shows decomposed median and IQR ellipses from Figure 3A in that closed-loop courses of *P*_S_ and *RR* are derived over the respiratory period with the normalized duration of 2π. In both cases, for supine (Figure 4A) and standing (Figure 4B), a delayed course of *RR* with respect to *P*_S_ is visible, an intrinsic property of the baroreflex with *P*_S_ acting as stimulus and *RR* representing a delayed response. The delay is significantly smaller for supine than standing (39.4° versus 41.3° for median courses) as confirmed by numerical data in Table 2, indicating again a more effective baroreflex in supine. The delay increases from the 25th to the 75th interquartile (Table 2).

**TABLE 2:** Characteristic parameters of the derived courses of *P*_S_ and *RR* out of the estimated median and IQR ellipses of spontaneous cardiovagal baroreflex cycles for supine and standing (Figure 4).

	Phase shift (°)		Deflection of *RR* (ms)		Deflection of *P*_S_ (mmHg)	
	Supine	Standing	Supine	Standing	Supine	Standing
25th quantile	27.3	29.8	45.4	35.1	5.2	7.7
Median	39.4	41.3	70.7	47.9	5.8	8.9
75th quantile	54.3	56.7	106.9	65.1	7.1	9.7

*All differences between supine and standing are statistically significant (p < 0.05).*

The peak-to-peak deflection of the median *P*_S_ over 2π is smaller for supine (5.8 mmHg with IQR 1.9 mmHg) than for standing (8.9 mmHg with IQR 2 mmHg), whereas the peak-to-peak deflection of the median *RR* is larger for supine (70.7 ms with IQR 61.5 ms) than for standing (47.9 ms with IQR 30 ms). This clearly indicates a higher *BRS* and, thus, an increased baroreflex efficiency for supine than standing and is in line with data from Figures 2A–D.

### Ellipse Parameters for Postural Changes

**FIGURE 5:**
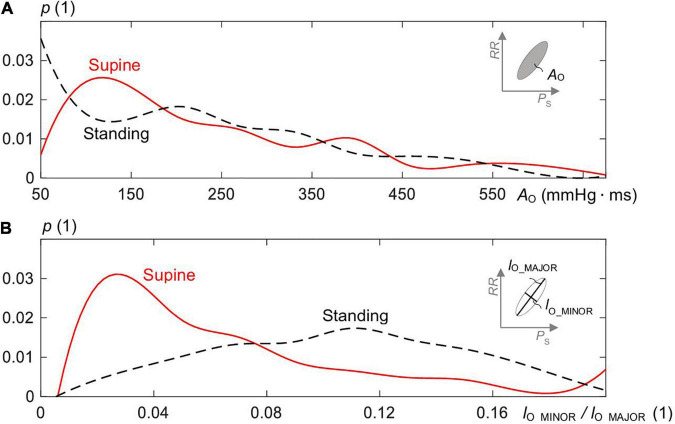
Distribution of **(A)** the area *A*_O_ of the estimated ellipses of the spontaneous CBC and **(B)** the ratio of minor to major axis length (=*l*_O_MINOR_/*l*_O_MAJOR_) for supine and standing with *p* as the probability of the distribution.

Figure 5 shows the distributions of *A*_O_ (Figure 5A) and the ratio *l*_O_MINOR_/*l*_O_MAJOR_ (Figure 5B) of the estimated overall ellipses for supine and standing. The median *A*_O_ for supine was 209 mmHg. ms (339 ± 516) and for standing 224 mmHg. ms (346 ± 527) with no statistical difference in between. However, for the ratio, there was a statistical difference (*p* < 0.005) with 0.11 (0.12 ± 0.8) for standing and 0.05 (0.07 ± 0.06) for supine, i.e., the ellipses for supine are significantly slimmer than for standing, which confirms the qualitative observation in Figure 3A.

**FIGURE 6:**
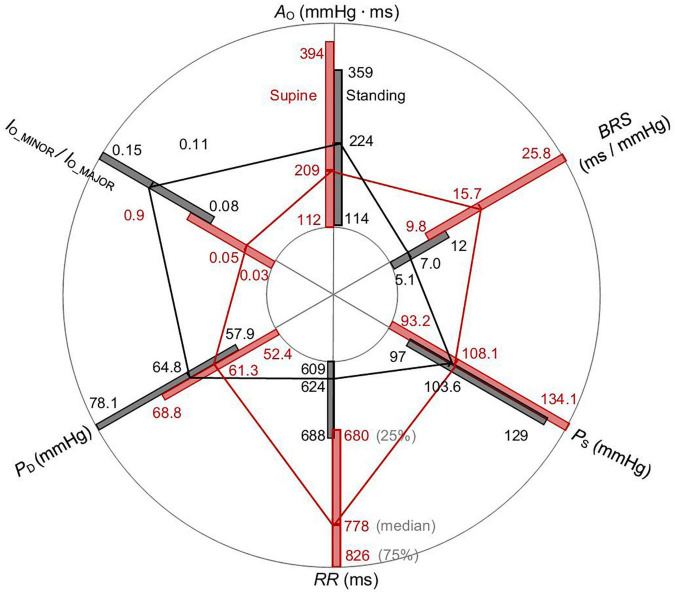
Radar plot of *P*_S_, *P*_*D*_, *RR*, *A*_O_, *BRS*, and the ratio *l*_O_MINOR_/*l*_O_MAJOR_ of the spontaneous CBC for supine and standing. Whereas medians of *RR* and *BRS* are elevated, *P*_*D*_ and the ratio *l*_O_MINOR_/*l*_O_MAJOR_ are declined for supine than standing. The values *A*_O_ and *P*_S_ do not significantly change for postural change.

The radar plot in Figure 6 summarizes *P*_S_, *P*_*D*_, *RR*, *A*_O_, *BRS* (for both up and down sequences), and the ratio *l*_O_MINOR_/*l*_O_MAJOR_ of the spontaneous CBCs for supine and standing using an image-based procedure. Each box length represents the 25th to 75th IQR with an indicated median value (see denoted axis for *RR* in Figure 6). The supine position shows significantly elevated *RR* and *BRS* and significantly depressed *P*_*D*_, *l*_O_MINOR_/*l*_O_MAJOR_ for supine in comparison with standing, whereas there is no significant difference found in *A*_O_ and *P*_*S.*_ In particular, the supine position with the median of 15.7 ms/mmHg shows a significantly higher *BRS* than standing with only 7 ms/mmHg. Likewise, the supine position has a larger *RR* as compared with standing (778 versus 624 ms).

**FIGURE 7:**
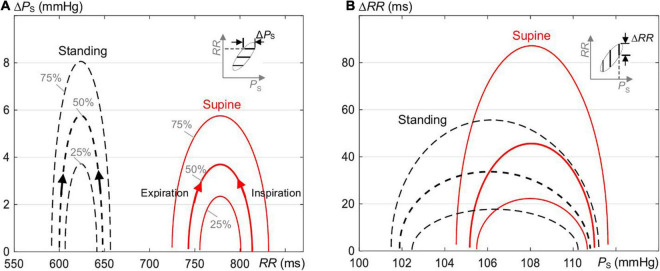
The magnitude of hysteresis loop (Figure 1C) in terms of changes **(A)** Δ*P*_S_ of *P*_S_ as a function of *RR* and **(B)** Δ*RR* of *RR* as a function of *P*_S_ of the estimated ellipses of the spontaneous CBC for supine and standing (Figure 3A). The magnitude along *P*_S_ is increased and along *RR* is reduced for standing as compared with supine.

Figure 7 illustrates that the baroreflex-related variability Δ*P*_S_ of *P*_S_ as a function of *RR* (Figure 7A) is reduced, whereas Δ*RR* of *RR* as a function of *P*_S_ (Figure 7B) is elevated for supine as compared with standing as a consequence of steeper ellipses in supine than standing (Figure 3A). The median magnitude of the formed hysteresis is about 50 ms and 3.7 mmHg for supine and about 32 ms and 5.8 mmHg for standing, in agreement with Figure 2C. For supine, this highlights a stronger baroreflex responses (in ms) at a given value of *P*_S_ and a stronger smoothing of blood pressure (in mmHg) at a given value of *RR*.

**FIGURE 8:**
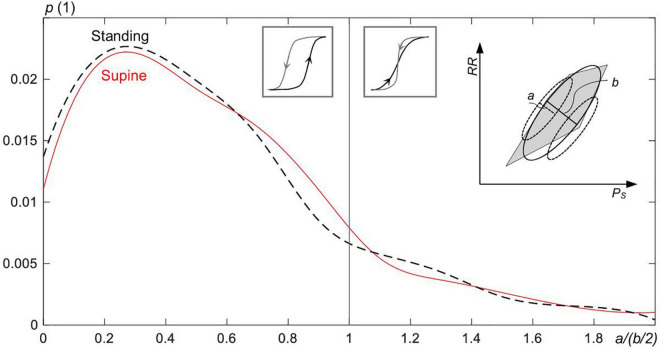
The probability *p* for the overlap of up and down sequences within the hysteresis of the estimated ellipses of the spontaneous CBC for supine and standing. The ratio *a*/(*b*/2)—for the definition, see the inset figure to the right—goes to zero the broader the width of the hysteresis (=*b*) and the more linear is the down sequence or, in analogy, the up sequence (linearity is inversely proportional to *a*). For *a*/(*b*/2) ≤ 1, the up and down sequences do not overlap; otherwise, sequences overlap with their strong fluctuations. Intersected sequences forming the hysteresis are less frequent (17.4% of all cases) than nonintersected sequences.

Figure 8 shows that the majority of CBC ellipses for supine and standing, respectively, are composed of nonoverlapping sequences. Namely, 83.4 and 81.8% of ellipses for supine and standing, respectively, show the ratio *a*/(*b*/2) ≤ 1.

## Discussion

The present work introduces a novel ellipse-based method to characterize hysteretic behavior of the spontaneous CBC as governed through spontaneous respiration and, thus, spontaneous pressure changes. The applicability and rationale of the method are shown for orthostatic tests when comparing supine and standing.

### Beat-to-Beat Analysis

The beat-to-beat analysis of Figures 1, 2 considers separately up and down sequences of spontaneous CBC (for expiration and inspiration, respectively) with individual *BRS* values, forming a base for ellipses (for the whole respiratory cycle) with its own *BRS*. An inverse relationship between beat-to-beat deflections in *P*_S_ and *RR* is clearly evident (Figure 2), and it highlights the rationale of the baroreflex to buffer changes in blood pressure at the cost of the heart-rate variability. The maximum deflection in *P*_S_ from one beat to the next can be seen at the onset of expiration (Figure 2A), where arterial vessels are maximally unloaded in the course of the respiration cycle (Figure 1C). In fact, barosensory vessels are more distensible for falling pressure than rising given relatively large changes in the pressure (Lénárd et al., 2000). Higher individual *BRS* result for supine than for standing (Figure 2E).

### Baroreflex Hysteresis – General Perspective

For expiration, both *P*_S_ and *RR* increase, whereas the baroreflex resets at the end of expiration and then lowers *P*_S_ and *RR* for inspiration. In particular, the last beat of each sequence forms an initial condition for the subsequent sequence (for baroreflex activity), namely, for its first beat. Consequently, the formed (median) ellipse of the spontaneous CBC with up and down sequences shows a particular hysteretic width and shape. Because more than 80% of the spontaneous CBC ellipses are composed of nonoverlapping sequences (Figure 8), it underscores even more a hysteresis behavior of CBC. Hystereses characteristics seem to be characteristic for supine and standing (Figure 3A), i.e., for the change in the working point of the baroreflex following changes in the static blood pressure in the course of an orthostatic test.

The hysteresis behavior of CBCs—or the width of the fitted ellipse of the cardiovagal CBC—is due to heterogenicity in nature, speed, and population of afferent baroreceptors (Seagard et al., 1990) in addition to nonlinear mechanics of barosensory vessels and neuronal mechanisms (Studinger et al., 2007). The fast myelinated A_β_ and A_δ_ fibers from baroreceptors are more sensitive to the pulsatile pressure change (Seagard et al., 1990; Rogers et al., 1993, 1996), whereas slow C fibers (and small A fibers) sense the absolute blood pressure changes. In addition, a relatively slow mechanical response of baro-sensory vessels (Bonyhay et al., 1997) and sympathovagal balance (Levy and Zieske, 1969) contributes to the hysteresis behavior of CBCs. The hystereses is also clearly favored by the observed delay between the *RR* and *P*_S_ courses (of about 40°; see Figure 4).

### Baroreflex Hysteresis – Supine Versus Standing

In supine, the median ellipse is steeper and more elongated (Figure 5B), indicating a higher baroreflex efficiency here. Namely, the proposed elliptic method shows that the median ellipse is elongated in the *RR* direction in supine with the significantly larger median θ_O_ of 85.8°, the significantly larger median deflection in *RR* of 70.7 ms (baroreflex output) for the significantly smaller median deflection in *P*_S_ of 5.8 mmHg (baroreflex input) as compared with standing with the respective values 81.5°, 47.9 ms, and 8.9 mmHg (Tables 1, 2). The resulting median values of *BRS* of the spontaneous CBC are significantly higher for supine with 15.7 ms/mmHg than standing with 7 ms/mmHg. The hysteresis area *A*_O_ shows no statistical differences between supine and standing; however, the ratio of minor to major axes *l*_O_MINOR_/*l*_O_MAJOR_ indicates a significantly slimmer ellipses for supine than standing. Therefore, the elliptic method uncovers that the hysteresis elongation of the spontaneous CBC is larger, whereas the associated hysteresis width is smaller for supine than standing. Likewise, the hysteresis magnitude along *RR* with the median of 50 ms for supine is larger than 32 ms for standing, whereas the reverse is true for *P*_S_, indicating a stronger smoothing action for supine (Figure 7).

The integrated *BRS* is reduced for standing due to a hypothesized reduction in the neural component of the baroreflex (Taylor et al., 2013). Namely, the gravitational pooling of blood within peripheral veins reduces the cardiac filling pressure, which, in turn, reduces the arterial blood pressure (Rowell, 1986; Nixon, 1988). The barosensory vessels become less inflated, whereas the afferent (vagal) activity of baroreceptors declines (Steinback et al., 2005; Taylor et al., 2013) and reduces the *BRS* of the spontaneous CBC in standing (Figure 3A) with damped respiratory sinus arrythmia (Faes et al., 2011). In addition, the autonomic nervous system increases the total periphery resistance in standing while accelerating the sympathetic outflow (Incognito et al., 2019); e.g., higher angles of the tilt table elevate the muscle sympathetic nerve activity (Cooke et al., 1999). This outflow seems to add more nonlinearity through the efferent sympathetic pathway (Wang et al., 1993; Ursino, 1998) to the hysteresis of the spontaneous CBC than for supine, resulting in a wider hysteresis for standing.

The center of the estimated ellipse moves for positional changes, which shape the overall sigmoid shape of the baroreflex with the moving working point from supine to standing (Figure 3A). The right wing of the overall hysteresis marks the rising blood pressure, whereas the left wing marks the falling blood pressure, in line with stronger baroreceptor activity for increasing blood pressure than decreasing at a given blood pressure value (Chapleau and Abboud, 1987; Chapleau et al., 1988; Rudas et al., 1999). The local hystereses of CBCs lose their steepness and widen for standing in comparison to supine with the aforementioned changes in the local inclination angle θ_O_ and the local *BRS*. In terms of quantitative analysis, the working point of the baroreflex decreases significantly from 778 ms for supine to 624 ms for standing, whereas there is no significant change in *P*_S_ (Figure 6). Although individual spontaneous CBCs illustrate cardiopulmonary baroreflex regulation with respiration as stimulus, the overall baroreflex shape illustrates baroreflex regulation with static blood pressure changes (stemming from positional changes) as stimulus for both arterial and cardiopulmonary baroreflex (Fadel and Raven, 2012). Likewise, whereas the spontaneous CBC corresponds rather to the linear portion of the overall hysteresis and is controlled predominantly by the parasympathetic system, the overall hysteresis shows a sigmoidal shape and is determined by the interplay of both parasympathetic (vagal) and sympathetic outflows (Chapleau and Abboud, 1987; Chapleau et al., 1988; Ursino, 1998).

Detailed analysis of up and down sequences forming CBCs (Supplementary Table 1 and Figure 2A) shows that up sequences tend to be steeper than down sequences for supine, whereas the reverse is true for standing. In fact, this difference in steepness tends to widen the formed hysteresis in the course of respiration. The larger width of hysteresis, such as in standing as compared with supine, underscores the reduced neural component of the baroreflex for standing than supine (Taylor et al., 2013), whereas the gain control is under the influence of both neural and mechanical components of baroreflex (Studinger et al., 2007).

The basic applicability of the elliptic method of CBC can be illustrated by considering spontaneous deflections of *P*_S_. This deflection for up sequences is about 4.3 and 7.3 mmHg for supine and standing (as medians), respectively, and for down sequences about 5.2 and 7.3 mmHg for supine and standing, respectively. Compared with this, the elliptic method confirms these differences with 5.8 and 8.9 mmHg for supine and standing, respectively (Table 2). The elliptic method offers also insights into the time domain (Figure 4) in that it discloses a delayed *RR* course with respect to the *P*_S_ course over the spontaneous breathing cycle. Namely, the courses of *P*_S_ and *RR* follow the same trend in rising and falling as governed by the *BRS*, and once the pressure direction changes, the course of *RR* still follows its trend with a median delay of about 40° for spontaneous breathing. The delay is qualitatively comparable with that during paced breathing at 0.1 and 0.25 Hz, showing the respective delays of 60° down to 0° (Cooke et al., 1999; Karemaker and De Boer, 2017). The observed delay by 39.4° for supine is less than 41.3° for standing (Table 2) for spontaneous breathing, indicating a seemingly increased baroreflex efficiency in supine and a reduced contribution of the slow sympathetic baroreflex arm (Cooke et al., 1999). However, it should be noted that not only baroreflex, but also respiratory sinus arrythmia itself affects the observed *RR* values, whereas the latter effect depends on the respiration rate and even reaches its maximum at 0.1 Hz (Angelone and Coulter, 1964). Thus, the perceived change in the baroreflex efficiency from standing to supine should be considered with caution.

For paced breathing at the median respiratory frequency of 0.2 Hz, the delay changed from 53° for 80° tilt down to −12° for supine (Cooke et al., 1999; Karemaker and De Boer, 2017), whereas our data for spontaneous breathing with the median respiratory frequency of 0.18 Hz (0.19 ± 0.03) and 0.21 Hz (0.21 ± 0.03) for respective supine and standing show a delay of about 40°. Thus, paced breathing in supine seems to show lower and even reversed delays (i.e., a noncausal relationship between blood pressure and heart rate) as compared with spontaneous breathing. The type of respiration and the respiration rate clearly affect the amount of *RR* changes, the relationship between *P*_S_ and *RR*, and a resulting change of θ_O_ (related to the delay amount). The delay is a result of the different *BRS* values for up and down sequences and the set-point changes of the baroreflex when going from up to down sequences (and vice versa) as well as different latencies of neural and mechanical arms (Bonyhay et al., 1997; Studinger et al., 2007) and the sympathovagal interplay (Masuda et al., 1984). In fact, Figure 4 shows how the mechanical and neural arms work in concert. Please note that only the neural arm is responsible for this set-point change during pressure direction changes (Studinger et al., 2007).

### Methodological Issues

In our study, spontaneous respiration served as a modulatory input to the arterial pressure to characterize the hysteretic behavior of the spontaneous CBC. Derived parameters of the resulting ellipses, each for a single respiratory cycle, are suggested to characterize the spontaneous variability of hysteresis and, thus, to characterize the spontaneous CBCs. The spontaneous CBC has an advantage of considering only respiration-related baroreflex function, minimizing influence of other cofactors impacting baroreflex function. Here, the duration of the recording is not limited by the duration of any artificial perturbating stimulus and, thus, allows long-term investigations of the cardiovascular system in (chronic) conditions other than (temporary) clinical settings.

We considered sequences of *P*_S_ and *RR* without any delay in between regardless of the actual level of heart rate to keep the number of valid respiratory-related cycles of CBCs as high as possible. However, authors in Pickering and Davies (1973) show that mutually undelayed sequences of *P*_S_ and *RR* correlate best for heart rate below 75 1/min, whereas for higher heart rates, the value of *P*_S_ corelates best with the next *RR* value.

Baroreflex sequences were already analyzed without differentiating them into up and down sequences and without any vasoactive pressure induction (Bertinieri et al., 1985; Steptoe and Vögele, 1990; Hughson et al., 1993; Choi et al., 2006), analyzed with their differentiation for postural changes including head-up tilt (De Maria et al., 2018, 2019), or for vasoactive pharmacological substances inducing pressure changes in a relatively wide range (such as the modified Oxford method; Studinger et al., 2007; Taylor et al., 2013). In contrast, the proposed closed-loop ellipse method not only differentiates up and down sequences, but also explicitly considers their natural succession over the course of the spontaneous respiration cycle without using any artificial stimulus. In particular, the analyzed respiration-related CBC reflects a natural baroreflex during natural posture changes in contrast to head-up tilt or other artificial stimuli. The proposed method and its derived parameters, such as inclination angle θ_O_, covers the neural and mechanical arms of the baroreflex without measuring the barosensory vessel diameters. In contrast to the state of the art, the considered closed-loop CBC has an advantage in considering pressure direction changes from expiration to inspiration and vice versa, thus considering the associated resetting of the baroreflex from one sequence to another (set-point changes). This yields a more robust estimation of the spontaneous cardiovagal hysteresis wings (Figure 1C).

Whereas, in the classical Oxford method, *P*_S_ is perturbated via pharmacological substances by >10% of the baseline *P*_S_ (usually by >15 mmHg; Taylor et al., 2013) to characterize the cardiovagal baroreflex, we use the spontaneous respiration-related perturbation of *P*_S_ in a smaller range of about 5.1 ± 3% (mean ± STD) of the baseline *P*_S_ for supine (5.6 ± 3.7 mmHg) and of about 7.1 ± 4% for standing (7.9 ± 4 mmHg); compare with Table 2. Thus, the proposed ellipse method characterizes the spontaneous CBCs without any pharmacological perturbations in the rather linear portion of baroreflex hysteresis with deflections of *P*_S_ in the approximate range of 7.9 ± 4 mmHg (Figure 3A).

Supplementary Table 1 compares *BRS* values for up and down sequences as estimated with the classical linear regression method and our proposed ellipse method, the latter being applied to individual up and down sequences as well as sequences composing CBC, see Supplementary Figure 2. There are no significant differences between both methods when considering up and down sequences, which is in line with De Maria et al. (2019) but contrasts with Eckberg and Sleight (1992) and Studinger et al. (2007). However, it can be observed in Supplementary Table 1 and Supplementary Figure 2 that sequences contributing to CBC (Supplementary Figure 2B) tend to show higher *BRS* than all sequences (Supplementary Figure 2A). In addition, the ellipse method on CBCs (Eq. 3) shows significantly higher median *BRS* for both supine and standing when compared with *BRS* for all sequences calculated with either the regression or ellipse method (Supplementary Table 1 and Supplementary Figure 2). This is because most of the up and down sequences (>80%; see Figure 8) do not overlap, their individual gains are usually different (with this difference even depending on the posture; see Supplementary Figure 2C), and the set-points of the baroreflex “jump” from up to down sequences (and vice versa) when the direction of the pressure changes, all of them affecting *BRS* of the whole CBC (Figure 1A). In particular, the first beat of expiration has the maximum deflection in *P*_S_ (Figure 2A) and a little change in *RR* (Figure 2C), which pushes more weight to the lower part of the CBC and, thus, aligns the resulting ellipse toward larger θ_O_ and, thus, larger *BRS* of the whole CBC.

The larger *BRS* of the whole CBC as well as larger *BRS* of sequences contributing to CBC (Supplementary Table 1) in comparison with *BRS* of individual up and down sequences may highlight the additional effects of the neural arm involved in the set-point change. This is because the individual sequences account for only both the mechanical and the neural arms acting within the single sequence.

In addition, it can be observed in Supplementary Table 1 that mean and median values strongly differ in some instances. This is due to frequent outliers that account for about 10% of all data points and, thus, affect the mean *BRS* but not the median *BRS*. The attained *BRS* using the proposed ellipse method were highly correlated by about 0.99 (*p* < 0.005) with *BRS* from the regression method in both supine and standing.

Because our analysis shows that about 17.4% of all up and down sequences forming CBC intersect with each other (Figure 8), whereas the Khachiyan algorithm (Kumar and Yildirim, 2005) cannot deal with intersected sequences, we focus on the image-based procedure. As an advantage, this procedure can consider both intersecting and nonintersecting sequences of CBCs.

### Limitations

**FIGURE 9:**
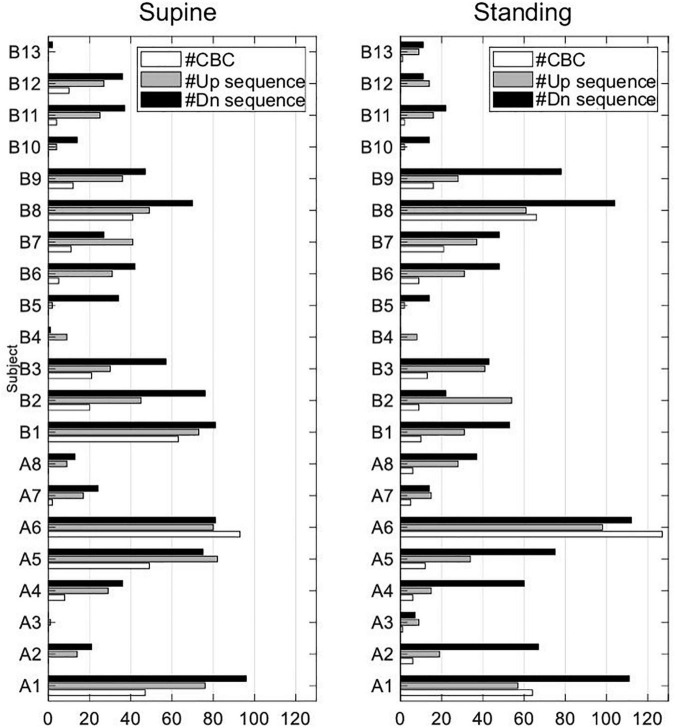
Absolute number of used up and down sequences and sequences forming CBCs in all subjects of EuroBaVar data set.

The data extraction from the EuroBaVar data set shows that only 3 out of 21 subjects (A1, A6, B8) contributed most of the up and down sequences, fulfilling the criteria to form a CBC in supine and standing; for absolute numbers, see Figure 9. Subject A5 mostly contributed to CBCs in supine. Sequences forming CBCs did not exist or were fewer than five in cases such as A3, B4, B5, B10, and B13. Although the analyzed subject pool is heterogenous, healthy subjects A1, A5, A6, and B8 seem to determine our results. This is because the observed results with healthy subjects yielded no statistical differences from those of all subjects. In addition, the relative number of outliers in the calculated *BRS* was quite high with about 10% and, thus, may have influenced the results, especially the observed differences between supine and standing. Because the proposed method uses consecutive up and down sequences with three or more beats to form CBC, i.e., in the course of the whole respiratory cycle, there must be six or more beats, high respiratory and slow cardiac rates might not meet these preconditions to form a proper CBC. In addition, weak dominance of the respiratory sinus arrhythmia, i.e., a weak modulation of the heart rate by respiration, may have influenced the detection of up and down sequences related to the respiration cycle. Because the respiration cycle was indirectly assessed from the respiratory sinus arrythmia and not directly measured, it may also influence our results. Here, paced breathing would have improved the formation and reliability of the formed CBC. The paced breathing could serve as a basis to differentiate respiration contribution (high-frequency components) from nonrespiratory components (low-frequency components) in terms of the frequency analysis to investigate the respiratory-related latencies during postural changes (Cooke et al., 1999; Karemaker and De Boer, 2017). Because the respiration rate affects *BRS* (Lehrer et al., 2003), the varying respiration rate may have also influenced our results. Moreover, our study characterizes the cardiovagal hysteresis for supine and standing only and not for extreme changes in blood pressure as would be necessary for the assessment of the complete baroreflex hysteresis.

There are also indications that tidal volume may affect cardiovagal *BRS* (Tzeng et al., 2009). However, as the tidal volume was not measured during the study, we can only assume that it had no influence on *BRS*, which is supported by the observation in Graf and Riedel (2017) that postural changes had no significant effects on the median tidal volume.

## Conclusion

A novel ellipse method is proposed to model and analyze spontaneous baroreflex sequences forming hysteresis during respiration and for changing posture from supine to standing. Gain and changes in set-points of sequences as well as the working point of baroreflex are considered with the method accounting for integrated mechanical and neural arms of the cardiovagal baroreflex. Observed differences between supine and standing offer instructive insights and provide a basis for future application of the proposed method in analyzing the arterial baroreflex under different conditions and stimuli.

## Data Availability Statement

The original contributions presented in the study are included in the article/Supplementary Material, further inquiries can be directed to the corresponding author/s.

## Ethics Statement

The studies involving human participants were reviewed and approved by Paris-Necker Committee. The patients/participants provided their written informed consent to participate in this study.

## Author Contributions

BD and EK conceived and designed the research, interpreted the results, and drafted and revised manuscript. BD and JB performed the data analysis, prepared the figures and tables. The authors agreed to be accountable for all aspects of the work in ensuring that questions related to the accuracy or integrity of any part of the work are appropriately investigated and resolved. All authors contributed to the article and approved the submitted version.

## Conflict of Interest

The authors declare that the research was conducted in the absence of any commercial or financial relationships that could be construed as a potential conflict of interest.

## Publisher’s Note

All claims expressed in this article are solely those of the authors and do not necessarily represent those of their affiliated organizations, or those of the publisher, the editors and the reviewers. Any product that may be evaluated in this article, or claim that may be made by its manufacturer, is not guaranteed or endorsed by the publisher.

## Footnotes

1
http://www.eurobavar.altervista.org

## Supplementary Material

Supplementary Figure 1 The spontaneous CBC with overlapping up and down sequences. **(A)** The up sequence (#1 to #4) intersects with the down sequence (♢1 to ♢3). **(B)** The generated binary image of the intersected sequences with the two separated regions and the respective subellipses (red) for each region. **(C)** The generated overall ellipse (bold black) out of subellipses (gray) as based on Eqs 1–3.

Supplementary Figure 2 The baroreflex sensitivity *BRS* assessed by the linear regression (LR) and the proposed ellipse (E) method for individual up and down sequences, CBC, and for supine and standing. **(A)** *BRS* of up sequences using LR and E methods, down sequences using LR and E methods (from the left to the right). **(B)** *BRS* of sequences forming CBC, up and down sequences using LR, up and down sequences using E. **(C)** *BRS* of up and down sequences using LR and E, *BRS* of CBC (Eq. 3). **(D)** *BRS* of up and down sequences forming CBC, LR versus E method.

Supplementary Table 1 Comparison of baroreflex sensitivity BRS values [median (mean ± standard deviation)] assessed by the linear regression and the proposed ellipse method for individual up and down sequences and for supine and standing. For comparison, *BRS* from the cardiovagal baroreflex cycles (CBC; Eq. 3) is provided, compare with Figure 6.
