# Supplementary figures and images for: Cardiovagal Baroreflex Hysteresis Using Ellipses in Response to Postural Changes
Source: Front Neurosci. 2021 Dec 9;15:720031. doi: 10.3389/fnins.2021.720031 (PMC8695984; doi:10.3389/fnins.2021.720031)

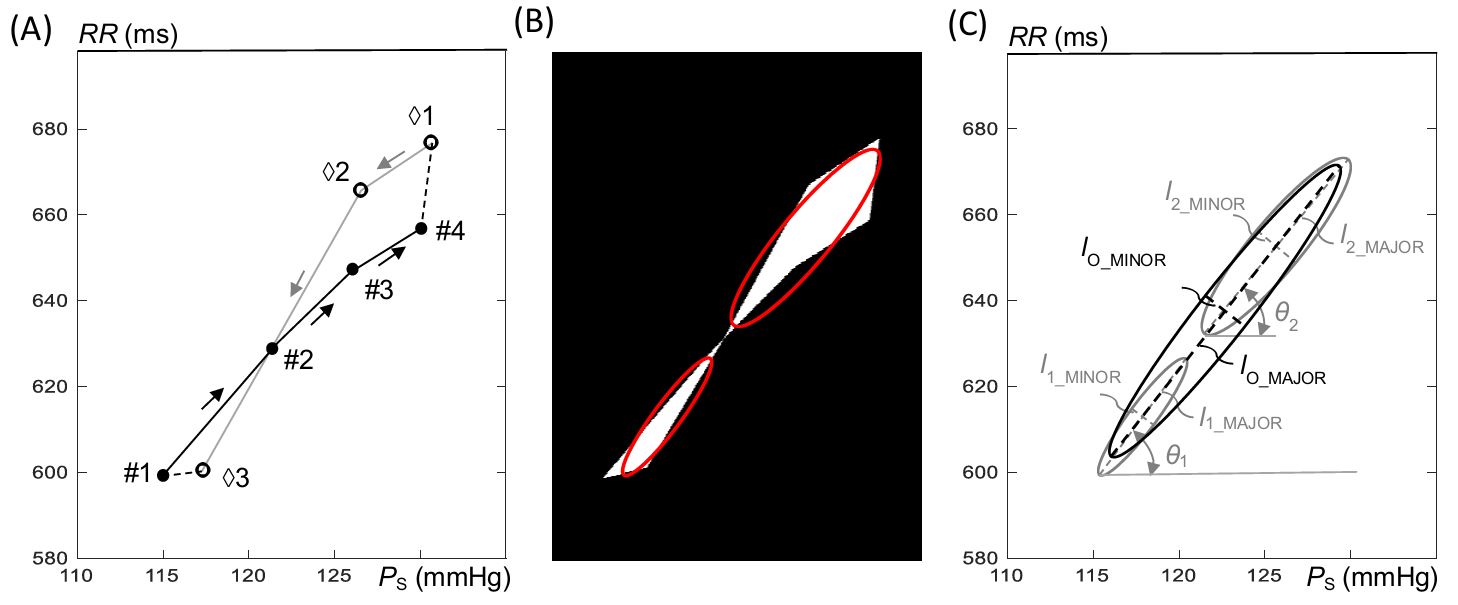

Supplement: Supplementary Figure 1 — The spontaneous CBC with overlapping up and down sequences. (A) The up sequence (#1 to #4) intersects with the down sequence (♢1 to ♢3). (B) The generated binary image of the intersected sequences with the two separated regions and the respective subellipses (red) for each region. (C) The generated overall ellipse (bold black) out of subellipses (gray) as based on Eqs 1–3. [file Image_1.jpg]

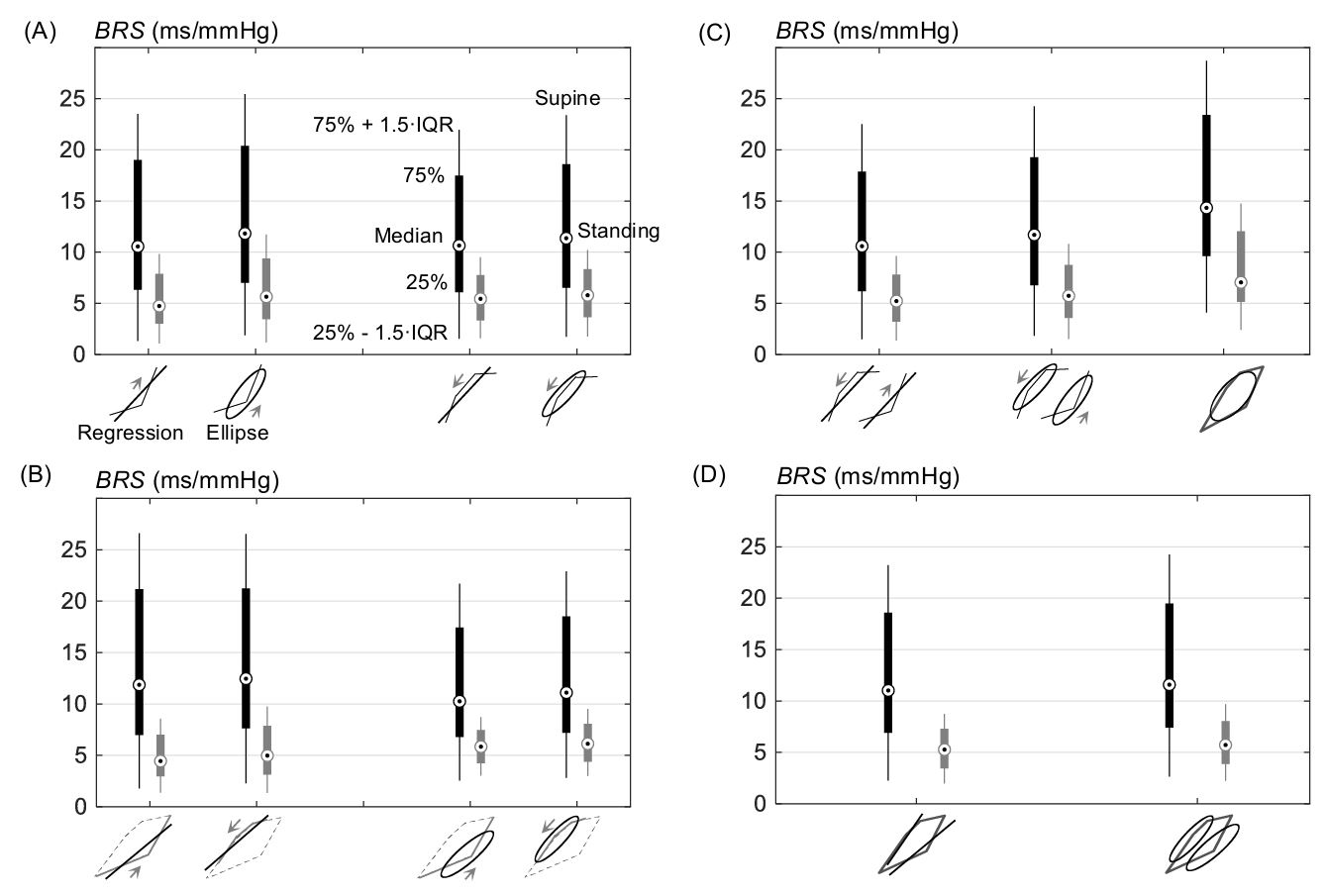

Supplement: Supplementary Figure 2 — The baroreflex sensitivity BRS assessed by the linear regression (LR) and the proposed ellipse (E) method for individual up and down sequences, CBC, and for supine and standing. (A) BRS of up sequences using LR and E methods, down sequences using LR and E methods (from the left to the right). (B) BRS of sequences forming CBC, up and down sequences using LR, up and down sequences using E. (C) BRS of up and down sequences using LR and E, BRS of CBC (Eq. 3). (D) BRS of up and down sequences forming CBC, LR versus E method. [file Image_2.jpg]
