# Supplementary material for: Cardiovagal Baroreflex Hysteresis Using Ellipses in Response to Postural Changes
Source: Front Neurosci. 2021 Dec 9;15:720031. doi: 10.3389/fnins.2021.720031 (PMC8695984; doi:10.3389/fnins.2021.720031)
Supplement: Supplementary Table 1 — Comparison of baroreflex sensitivity BRS values [median (mean ± standard deviation)] assessed by the linear regression and the proposed ellipse method for individual up and down sequences and for supine and standing. For comparison, BRS from the cardiovagal baroreflex cycles (CBC; Eq. 3) is provided, compare with Figure 6. [file Table_1.docx]

|  | | **Number of sequences** (supine, standing) | ***BRS* in supine  (**ms / mmHg**)** |  | ***BRS* in standing (**ms / mmHg**)** |  | ***p* value** | |
| --- | --- | --- | --- | --- | --- | --- | --- | --- |
| Regression | Up-sequences | 680, 609 | 10.5 (16.9 ± 42.6) |  | 4.7 (6.8 ± 7.0) |  | < 0.005 | |
|  | Down-sequences | 870, 951 | 10.6 (13.3 ± 11.9) |  | 5.4 (6.8 ± 7.1) |  | < 0.005 | |
|  | All sequences | 1550, 1560 | 10.6 (14.8 ± 29.6) |  | 5.2 (6.8 ± 7.0) |  | < 0.005 | |
|  | Up-sequences of CBC | 330, 286 | 11.9 (17.3 ± 16.9) |  | 4.4 (6.0 ± 6.3) |  | < 0.005 | |
|  | Down-sequences of CBC | 331, 312 | 10.3 (13.2 ± 12.1) |  | 5.8 (6.6 ± 4.7) |  | < 0.005 | |
|  | All sequences of CBC | 661, 598 | 11.0 (15.2 ± 14.8) |  | 5.3 (6.3 ± 5.5) |  | < 0.005 | |
| Ellipse | Up-sequences | 631, 575 | 11.5 (16.2 ± 17.0) |  | 5.1 (7.4 ± 7.2) |  | < 0.005 | |
|  | Down-sequences | 809, 908 | 11.2 (13.9 ± 11.9) |  | 5.7 (7.1 ± 8.3) |  | < 0.005 | |
|  | All sequences | 1440, 1483 | 11.3 (14.9 ± 14.4) |  | 5.6 (7.2 ± 7.9) |  | < 0.005 | |
|  | Up-sequences of CBC | 298, 277 | 12.4 (17.9 ± 17.9) |  | 5.0 (6.8 ± 7.0) |  | < 0.005 | |
|  | Down-sequences of CBC | 310, 305 | 11.1 (13.4 ± 9.4) |  | 6.1 (7.2 ± 5.1) |  | < 0.005 | |
|  | All sequences of CBC | 608, 582 | 11.6 (15.6 ± 14.4) |  | 5.2 (7.0 ± 6.1) |  | < 0.005 | |
|  | CBC (Eq. 3) | 386, 374 | 15.7 (29.6 ± 149) |  | 7.0 (12.8 ± 25.5) |  | < 0.005 | |
|  | | | | | | | |  |
|  | | | | | | | |  |
